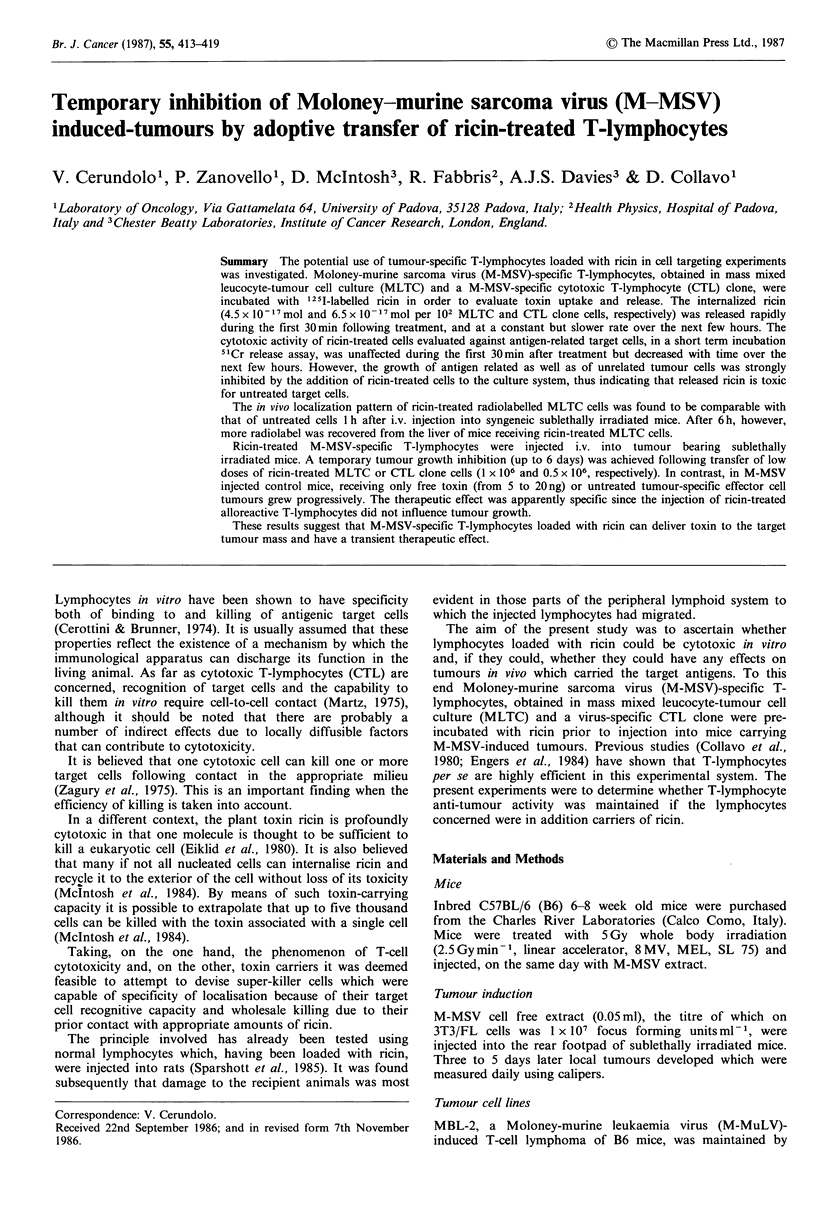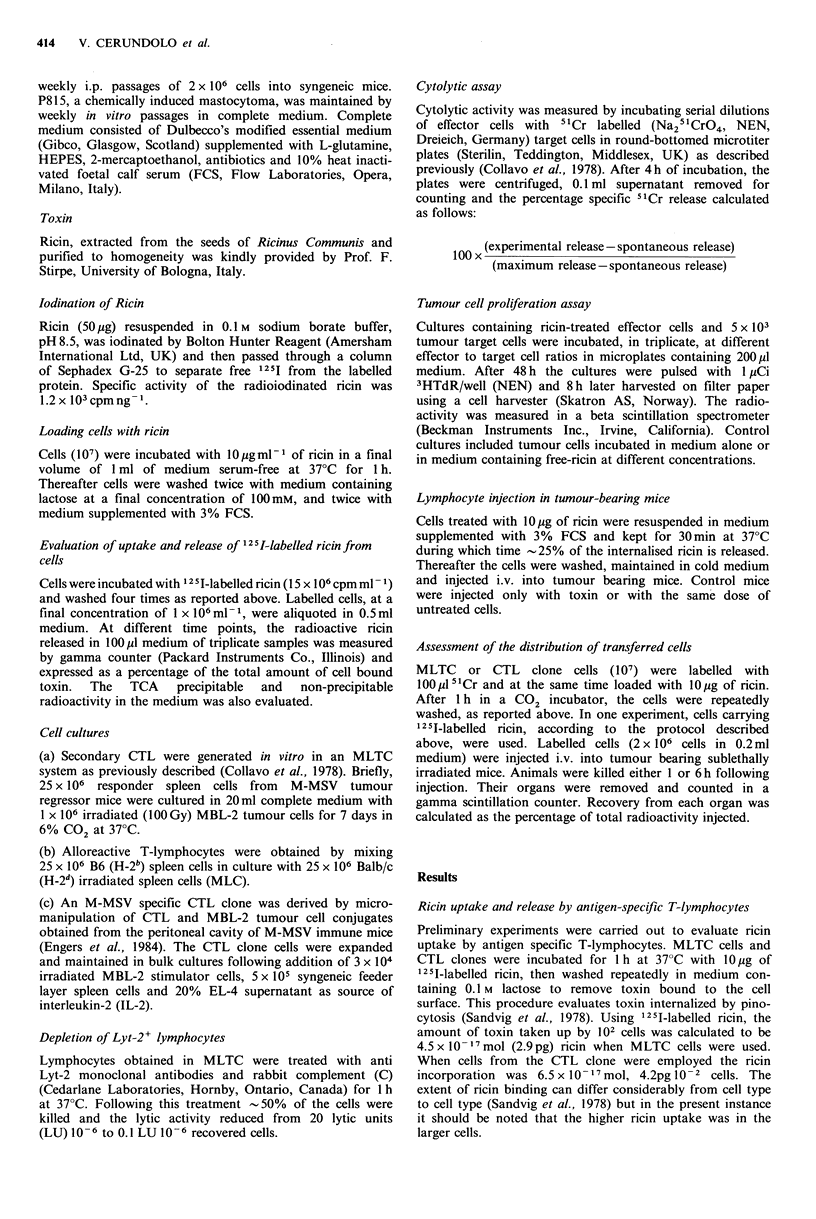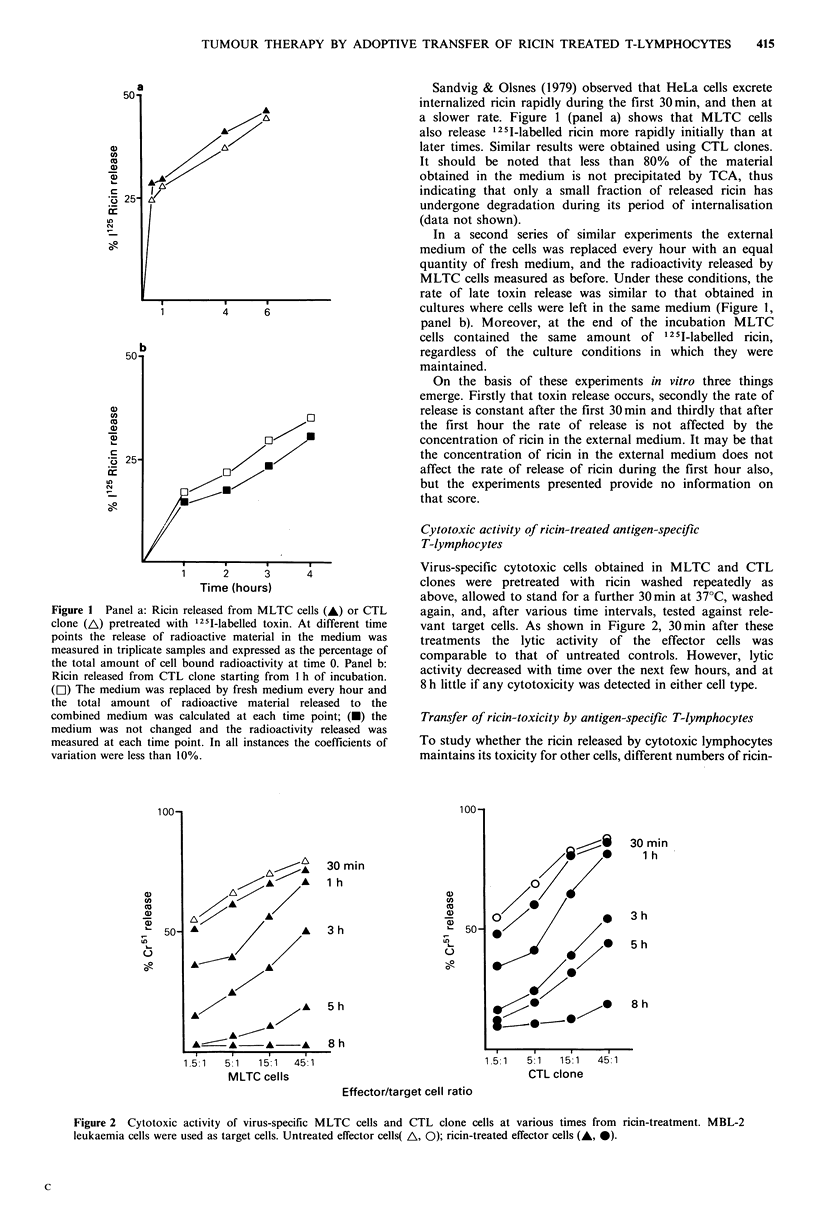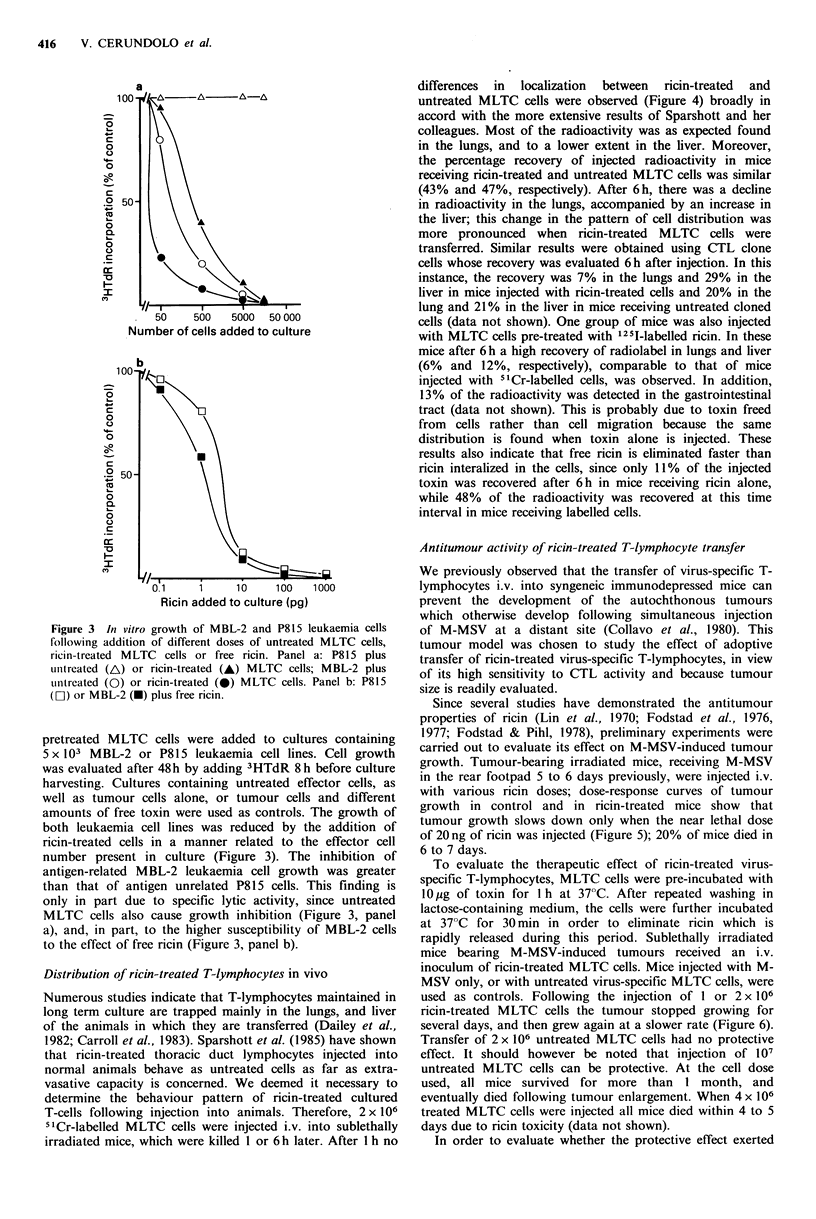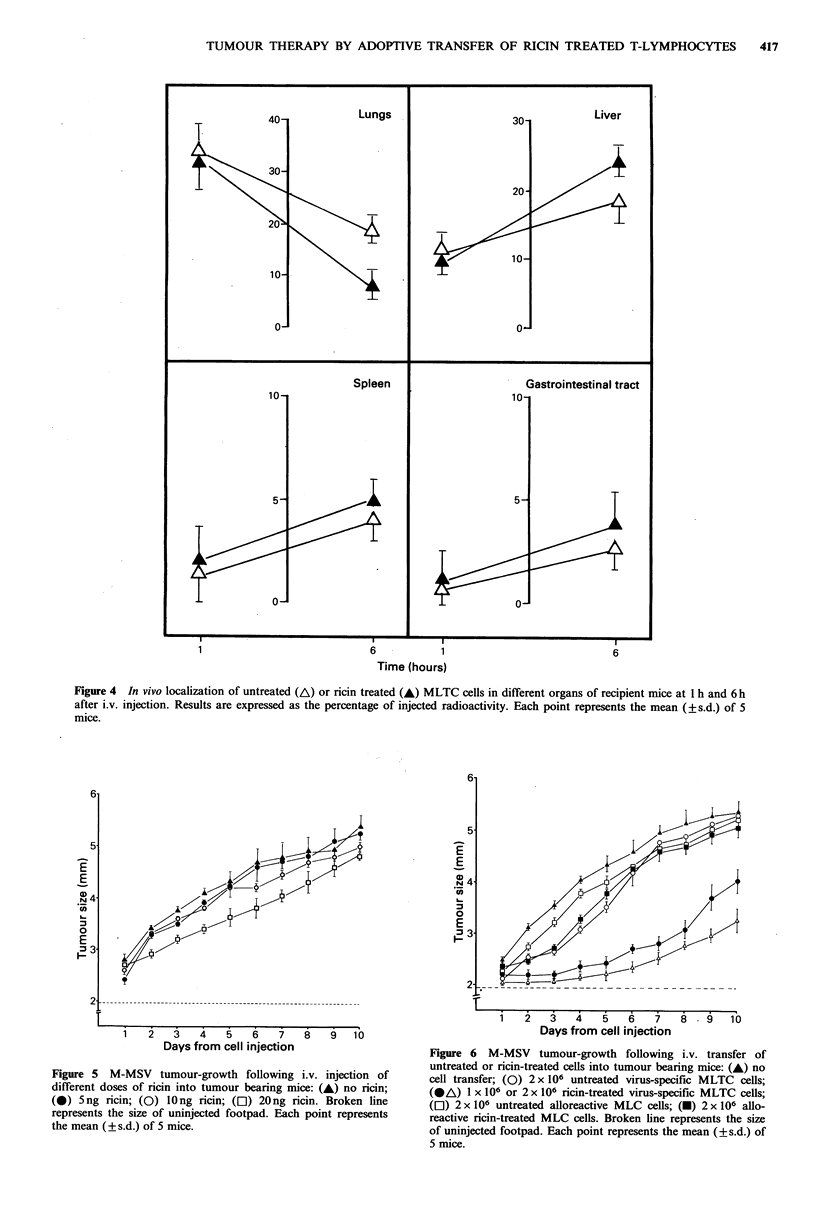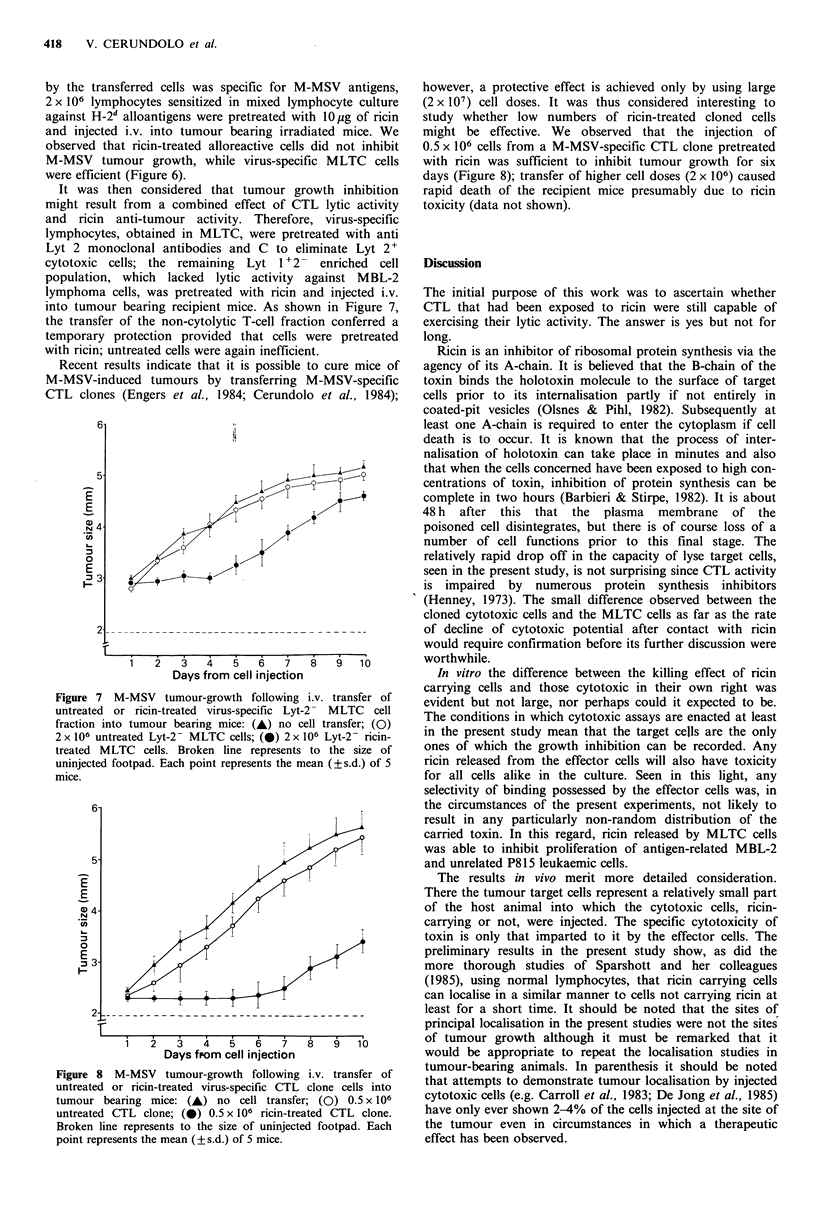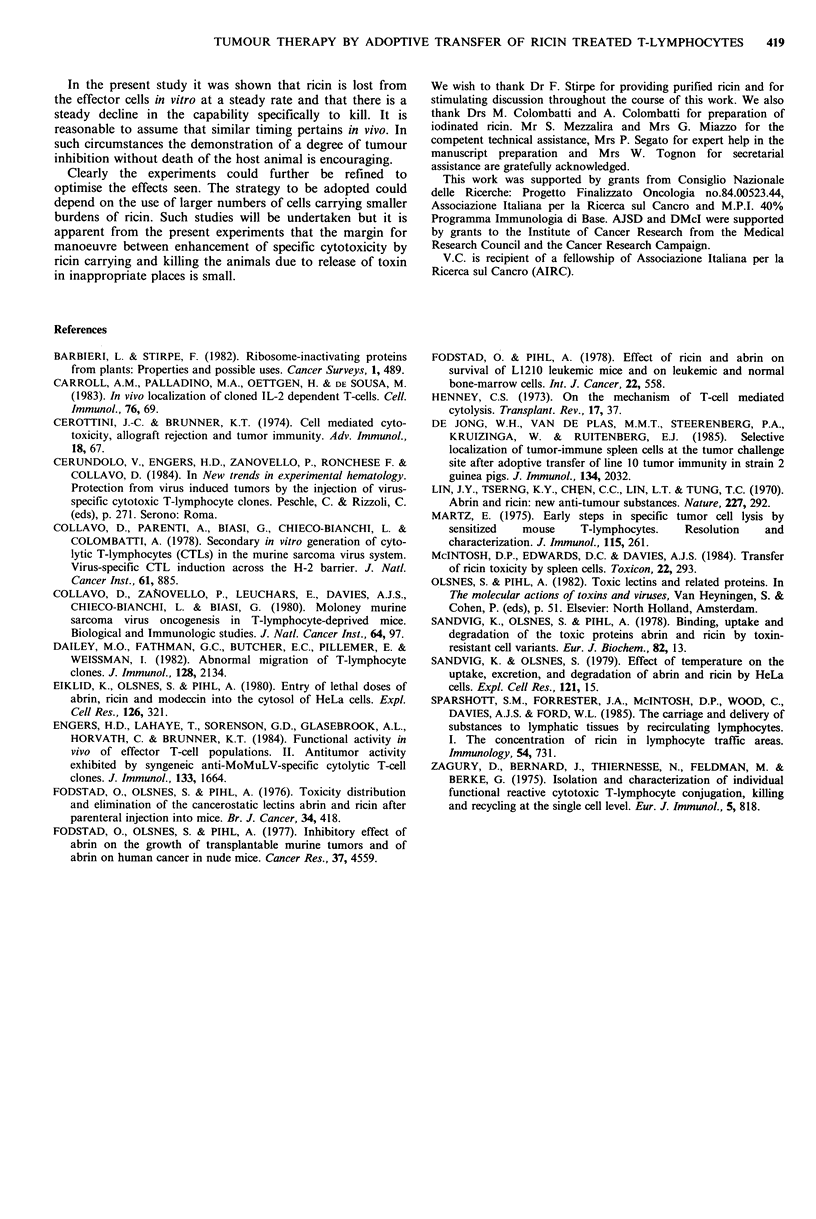# Temporary inhibition of Moloney-murine sarcoma virus (M-MSV) induced-tumours by adoptive transfer of ricin-treated T-lymphocytes.

**DOI:** 10.1038/bjc.1987.81

**Published:** 1987-04

**Authors:** V. Cerundolo, P. Zanovello, D. McIntosh, R. Fabbris, A. J. Davies, D. Collavo

## Abstract

The potential use of tumour-specific T-lymphocytes loaded with ricin in cell targeting experiments was investigated. Moloney-murine sarcoma virus (M-MSV)-specific T-lymphocytes, obtained in mass mixed leucocyte-tumour cell culture (MLTC) and a M-MSV-specific cytotoxic T-lymphocyte (CTL) clone, were incubated with 125I-labelled ricin in order to evaluate toxin uptake and release. The internalized ricin (4.5 X 10(-17) mol and 6.5 X 10(-17) mol per 10(2) MLTC and CTL clone cells, respectively) was released rapidly during the first 30 min following treatment, and at a constant but slower rate over the next few hours. The cytotoxic activity of ricin-treated cells evaluated against antigen-related target cells, in a short term incubation 51Cr release assay, was unaffected during the first 30 min after treatment but decreased with time over the next few hours. However, the growth of antigen related as well as of unrelated tumour cells was strongly inhibited by the addition of ricin-treated cells to the culture system, thus indicating that released ricin is toxic for untreated target cells. The in vivo localization pattern of ricin-treated radiolabelled MLTC cells was found to be comparable with that of untreated cells 1 h after i.v. injection into syngeneic sublethally irradiated mice. After 6 h, however, more radiolabel was recovered from the liver of mice receiving ricin-treated MLTC cells. Ricin-treated M-MSV-specific T-lymphocytes were injected i.v. into tumour bearing sublethally irradiated mice. A temporary tumour growth inhibition (up to 6 days) was achieved following transfer of low doses of ricin-treated MLTC or CTL clone cells (1 X 10(6) and 0.5 X 10(6), respectively). In contrast, in M-MSV injected control mice, receiving only free toxin (from 5 to 20 ng) or untreated tumour-specific effector cell tumours grew progressively. The therapeutic effect was apparently specific since the injection of ricin-treated alloreactive T-lymphocytes did not influence tumour growth. These results suggest that M-MSV-specific T-lymphocytes loaded with ricin can deliver toxin to the target tumour mass and have a transient therapeutic effect.


Br. J. Cancer (1987), 55, 413–419 © The Macmillan Press Ltd., 1987

### Temporary inhibition of Moloney–murine sarcoma virus (M–MSV) induced-tumours by adoptive transfer of ricin-treated T-lymphocytes

V. Cerundolo[1], P. Zanovello[1], D. McIntosh[3], R. Fabbris[2], A.J.S. Davies[3] & D. Collavo[1]

[1]*Laboratory of Oncology, Via Gattamelata 64, University of Padova, 35128 Padova, Italy;* [2]*Health Physics, Hospital of Padova, Italy and* [3]*Chester Beatty Laboratories, Institute of Cancer Research, London, England.*

**Summary** The potential use of tumour-specific T-lymphocytes loaded with ricin in cell targeting experiments was investigated. Moloney-murine sarcoma virus (M-MSV)-specific T-lymphocytes, obtained in mass mixed leucocyte-tumour cell culture (MLTC) and a M-MSV-specific cytotoxic T-lymphocyte (CTL) clone, were incubated with $^{125}$I-labelled ricin in order to evaluate toxin uptake and release. The internalized ricin ($4.5 \times 10^{-17}$ mol and $6.5 \times 10^{-17}$ mol per $10^2$ MLTC and CTL clone cells, respectively) was released rapidly during the first 30 min following treatment, and at a constant but slower rate over the next few hours. The cytotoxic activity of ricin-treated cells evaluated against antigen-related target cells, in a short term incubation $^{51}$Cr release assay, was unaffected during the first 30 min after treatment but decreased with time over the next few hours. However, the growth of antigen related as well as of unrelated tumour cells was strongly inhibited by the addition of ricin-treated cells to the culture system, thus indicating that released ricin is toxic for untreated target cells.

The *in vivo* localization pattern of ricin-treated radiolabelled MLTC cells was found to be comparable with that of untreated cells 1 h after i.v. injection into syngeneic sublethally irradiated mice. After 6 h, however, more radiolabel was recovered from the liver of mice receiving ricin-treated MLTC cells.

Ricin-treated M-MSV-specific T-lymphocytes were injected i.v. into tumour bearing sublethally irradiated mice. A temporary tumour growth inhibition (up to 6 days) was achieved following transfer of low doses of ricin-treated MLTC or CTL clone cells ($1 \times 10^6$ and $0.5 \times 10^6$, respectively). In contrast, in M-MSV injected control mice, receiving only free toxin (from 5 to 20 ng) or untreated tumour-specific effector cell tumours grew progressively. The therapeutic effect was apparently specific since the injection of ricin-treated alloreactive T-lymphocytes did not influence tumour growth.

These results suggest that M-MSV-specific T-lymphocytes loaded with ricin can deliver toxin to the target tumour mass and have a transient therapeutic effect.

Lymphocytes *in vitro* have been shown to have specificity both of binding to and killing of antigenic target cells (Cerottini & Brunner, 1974). It is usually assumed that these properties reflect the existence of a mechanism by which the immunological apparatus can discharge its function in the living animal. As far as cytotoxic T-lymphocytes (CTL) are concerned, recognition of target cells and the capability to kill them *in vitro* require cell-to-cell contact (Martz, 1975), although it should be noted that there are probably a number of indirect effects due to locally diffusible factors that can contribute to cytotoxicity.

It is believed that one cytotoxic cell can kill one or more target cells following contact in the appropriate milieu (Zagury *et al.*, 1975). This is an important finding when the efficiency of killing is taken into account.

In a different context, the plant toxin ricin is profoundly cytotoxic in that one molecule is thought to be sufficient to kill a eukaryotic cell (Eiklid *et al.*, 1980). It is also believed that many if not all nucleated cells can internalise ricin and recycle it to the exterior of the cell without loss of its toxicity (McIntosh *et al.*, 1984). By means of such toxin-carrying capacity it is possible to extrapolate that up to five thousand cells can be killed with the toxin associated with a single cell (McIntosh *et al.*, 1984).

Taking, on the one hand, the phenomenon of T-cell cytotoxicity and, on the other, toxin carriers it was deemed feasible to attempt to devise super-killer cells which were capable of specificity of localisation because of their target cell recognitive capacity and wholesale killing due to their prior contact with appropriate amounts of ricin.

The principle involved has already been tested using normal lymphocytes which, having been loaded with ricin, were injected into rats (Sparshott *et al.*, 1985). It was found subsequently that damage to the recipient animals was most evident in those parts of the peripheral lymphoid system to which the injected lymphocytes had migrated.

The aim of the present study was to ascertain whether lymphocytes loaded with ricin could be cytotoxic *in vitro* and, if they could, whether they could have any effects on tumours *in vivo* which carried the target antigens. To this end Moloney-murine sarcoma virus (M-MSV)-specific T-lymphocytes, obtained in mass mixed leucocyte-tumour cell culture (MLTC) and a virus-specific CTL clone were pre-incubated with ricin prior to injection into mice carrying M-MSV-induced tumours. Previous studies (Collavo *et al.*, 1980; Engers *et al.*, 1984) have shown that T-lymphocytes *per se* are highly efficient in this experimental system. The present experiments were to determine whether T-lymphocyte anti-tumour activity was maintained if the lymphocytes concerned were in addition carriers of ricin.

### Materials and Methods

### *Mice*

Inbred C57BL/6 (B6) 6–8 week old mice were purchased from the Charles River Laboratories (Calco Como, Italy). Mice were treated with 5 Gy whole body irradiation (2.5 Gy min$^{-1}$, linear accelerator, 8 MV, MEL, SL 75) and injected, on the same day with M-MSV extract.

### *Tumour induction*

M-MSV cell free extract (0.05 ml), the titre of which on 3T3/FL cells was $1 \times 10^7$ focus forming units ml$^{-1}$, were injected into the rear footpad of sublethally irradiated mice. Three to 5 days later local tumours developed which were measured daily using calipers.

### *Tumour cell lines*

MBL-2, a Moloney-murine leukaemia virus (M-MuLV)-induced T-cell lymphoma of B6 mice, was maintained by

Correspondence: V. Cerundolo.
Received 22nd September 1986; and in revised form 7th November 1986.

weekly i.p. passages of $2 \times 10^6$ cells into syngeneic mice. P815, a chemically induced mastocytoma, was maintained by weekly *in vitro* passages in complete medium. Complete medium consisted of Dulbecco's modified essential medium (Gibco, Glasgow, Scotland) supplemented with L-glutamine, HEPES, 2-mercaptoethanol, antibiotics and 10% heat inactivated foetal calf serum (FCS, Flow Laboratories, Opera, Milano, Italy).

*Toxin*

Ricin, extracted from the seeds of *Ricinus Communis* and purified to homogeneity was kindly provided by Prof. F. Stirpe, University of Bologna, Italy.

*Iodination of Ricin*

Ricin (50 $\mu$g) resuspended in 0.1 M sodium borate buffer, pH 8.5, was iodinated by Bolton Hunter Reagent (Amersham International Ltd, UK) and then passed through a column of Sephadex G-25 to separate free $^{125}I$ from the labelled protein. Specific activity of the radioiodinated ricin was $1.2 \times 10^3$ cpm $ng^{-1}$.

*Loading cells with ricin*

Cells ($10^7$) were incubated with 10 $\mu g\,ml^{-1}$ of ricin in a final volume of 1 ml of medium serum-free at 37°C for 1 h. Thereafter cells were washed twice with medium containing lactose at a final concentration of 100 mM, and twice with medium supplemented with 3% FCS.

*Evaluation of uptake and release of $^{125}I$-labelled ricin from cells*

Cells were incubated with $^{125}I$-labelled ricin ($15 \times 10^6$ cpm $ml^{-1}$) and washed four times as reported above. Labelled cells, at a final concentration of $1 \times 10^6\,ml^{-1}$, were aliquoted in 0.5 ml medium. At different time points, the radioactive ricin released in 100 $\mu$l medium of triplicate samples was measured by gamma counter (Packard Instruments Co., Illinois) and expressed as a percentage of the total amount of cell bound toxin. The TCA precipitable and non-precipitable radioactivity in the medium was also evaluated.

*Cell cultures*

(a) Secondary CTL were generated *in vitro* in an MLTC system as previously described (Collavo *et al.*, 1978). Briefly, $25 \times 10^6$ responder spleen cells from M-MSV tumour regressor mice were cultured in 20 ml complete medium with $1 \times 10^6$ irradiated (100 Gy) MBL-2 tumour cells for 7 days in 6% $CO_2$ at 37°C.

(b) Alloreactive T-lymphocytes were obtained by mixing $25 \times 10^6$ B6 (H-$2^b$) spleen cells in culture with $25 \times 10^6$ Balb/c (H-$2^d$) irradiated spleen cells (MLC).

(c) An M-MSV specific CTL clone was derived by micromanipulation of CTL and MBL-2 tumour cell conjugates obtained from the peritoneal cavity of M-MSV immune mice (Engers *et al.*, 1984). The CTL clone cells were expanded and maintained in bulk cultures following addition of $3 \times 10^4$ irradiated MBL-2 stimulator cells, $5 \times 10^5$ syngeneic feeder layer spleen cells and 20% EL-4 supernatant as source of interleukin-2 (IL-2).

*Depletion of Lyt-$2^+$ lymphocytes*

Lymphocytes obtained in MLTC were treated with anti Lyt-2 monoclonal antibodies and rabbit complement (C) (Cedarlane Laboratories, Hornby, Ontario, Canada) for 1 h at 37°C. Following this treatment ~50% of the cells were killed and the lytic activity reduced from 20 lytic units (LU) $10^{-6}$ to 0.1 LU $10^{-6}$ recovered cells.

*Cytolytic assay*

Cytolytic activity was measured by incubating serial dilutions of effector cells with $^{51}Cr$ labelled ($Na_2{}^{51}CrO_4$, NEN, Dreieich, Germany) target cells in round-bottomed microtiter plates (Sterilin, Teddington, Middlesex, UK) as described previously (Collavo *et al.*, 1978). After 4 h of incubation, the plates were centrifuged, 0.1 ml supernatant removed for counting and the percentage specific $^{51}Cr$ release calculated as follows:

$$100 \times \frac{(\text{experimental release} - \text{spontaneous release})}{(\text{maximum release} - \text{spontaneous release})}$$

*Tumour cell proliferation assay*

Cultures containing ricin-treated effector cells and $5 \times 10^3$ tumour target cells were incubated, in triplicate, at different effector to target cell ratios in microplates containing 200 $\mu$l medium. After 48 h the cultures were pulsed with 1 $\mu$Ci $^3$HTdR/well (NEN) and 8 h later harvested on filter paper using a cell harvester (Skatron AS, Norway). The radioactivity was measured in a beta scintillation spectrometer (Beckman Instruments Inc., Irvine, California). Control cultures included tumour cells incubated in medium alone or in medium containing free-ricin at different concentrations.

*Lymphocyte injection in tumour-bearing mice*

Cells treated with 10 $\mu$g of ricin were resuspended in medium supplemented with 3% FCS and kept for 30 min at 37°C during which time ~25% of the internalised ricin is released. Thereafter the cells were washed, maintained in cold medium and injected i.v. into tumour bearing mice. Control mice were injected only with toxin or with the same dose of untreated cells.

*Assessment of the distribution of transferred cells*

MLTC or CTL clone cells ($10^7$) were labelled with 100 $\mu$l $^{51}Cr$ and at the same time loaded with 10 $\mu$g of ricin. After 1 h in a $CO_2$ incubator, the cells were repeatedly washed, as reported above. In one experiment, cells carrying $^{125}I$-labelled ricin, according to the protocol described above, were used. Labelled cells ($2 \times 10^6$ cells in 0.2 ml medium) were injected i.v. into tumour bearing sublethally irradiated mice. Animals were killed either 1 or 6 h following injection. Their organs were removed and counted in a gamma scintillation counter. Recovery from each organ was calculated as the percentage of total radioactivity injected.

### Results

*Ricin uptake and release by antigen-specific T-lymphocytes*

Preliminary experiments were carried out to evaluate ricin uptake by antigen specific T-lymphocytes. MLTC cells and CTL clones were incubated for 1 h at 37°C with 10 $\mu$g of $^{125}I$-labelled ricin, then washed repeatedly in medium containing 0.1 M lactose to remove toxin bound to the cell surface. This procedure evaluates toxin internalized by pinocytosis (Sandvig *et al.*, 1978). Using $^{125}I$-labelled ricin, the amount of toxin taken up by $10^2$ cells was calculated to be $4.5 \times 10^{-17}$ mol (2.9 pg) ricin when MLTC cells were used. When cells from the CTL clone were employed the ricin incorporation was $6.5 \times 10^{-17}$ mol, 4.2 pg $10^{-2}$ cells. The extent of ricin binding can differ considerably from cell type to cell type (Sandvig *et al.*, 1978) but in the present instance it should be noted that the higher ricin uptake was in the larger cells.

**Figure 1** Panel a: Ricin released from MLTC cells (▲) or CTL clone (△) pretreated with $^{125}$I-labelled toxin. At different time points the release of radioactive material in the medium was measured in triplicate samples and expressed as the percentage of the total amount of cell bound radioactivity at time 0. Panel b: Ricin released from CTL clone starting from 1 h of incubation. (□) The medium was replaced by fresh medium every hour and the total amount of radioactive material released to the combined medium was calculated at each time point; (■) the medium was not changed and the radioactivity released was measured at each time point. In all instances the coefficients of variation were less than 10%.

Sandvig & Olsnes (1979) observed that HeLa cells excrete internalized ricin rapidly during the first 30 min, and then at a slower rate. Figure 1 (panel a) shows that MLTC cells also release $^{125}$I-labelled ricin more rapidly initially than at later times. Similar results were obtained using CTL clones. It should be noted that less than 80% of the material obtained in the medium is not precipitated by TCA, thus indicating that only a small fraction of released ricin has undergone degradation during its period of internalisation (data not shown).

In a second series of similar experiments the external medium of the cells was replaced every hour with an equal quantity of fresh medium, and the radioactivity released by MLTC cells measured as before. Under these conditions, the rate of late toxin release was similar to that obtained in cultures where cells were left in the same medium (Figure 1, panel b). Moreover, at the end of the incubation MLTC cells contained the same amount of $^{125}$I-labelled ricin, regardless of the culture conditions in which they were maintained.

On the basis of these experiments *in vitro* three things emerge. Firstly that toxin release occurs, secondly the rate of release is constant after the first 30 min and thirdly that after the first hour the rate of release is not affected by the concentration of ricin in the external medium. It may be that the concentration of ricin in the external medium does not affect the rate of release of ricin during the first hour also, but the experiments presented provide no information on that score.

### *Cytotoxic activity of ricin-treated antigen-specific T-lymphocytes*

Virus-specific cytotoxic cells obtained in MLTC and CTL clones were pretreated with ricin washed repeatedly as above, allowed to stand for a further 30 min at 37°C, washed again, and, after various time intervals, tested against relevant target cells. As shown in Figure 2, 30 min after these treatments the lytic activity of the effector cells was comparable to that of untreated controls. However, lytic activity decreased with time over the next few hours, and at 8 h little if any cytotoxicity was detected in either cell type.

### *Transfer of ricin-toxicity by antigen-specific T-lymphocytes*

To study whether the ricin released by cytotoxic lymphocytes maintains its toxicity for other cells, different numbers of ricin-

**Figure 2** Cytotoxic activity of virus-specific MLTC cells and CTL clone cells at various times from ricin-treatment. MBL-2 leukaemia cells were used as target cells. Untreated effector cells( △, ○); ricin-treated effector cells (▲, ●).

**Figure 3** *In vitro* growth of MBL-2 and P815 leukaemia cells following addition of different doses of untreated MLTC cells, ricin-treated MLTC cells or free ricin. Panel a: P815 plus untreated (△) or ricin-treated (▲) MLTC cells; MBL-2 plus untreated (○) or ricin-treated (●) MLTC cells. Panel b: P815 (□) or MBL-2 (■) plus free ricin.

pretreated MLTC cells were added to cultures containing $5 \times 10^3$ MBL-2 or P815 leukaemia cell lines. Cell growth was evaluated after 48h by adding $^3$HTdR 8h before culture harvesting. Cultures containing untreated effector cells, as well as tumour cells alone, or tumour cells and different amounts of free toxin were used as controls. The growth of both leukaemia cell lines was reduced by the addition of ricin-treated cells in a manner related to the effector cell number present in culture (Figure 3). The inhibition of antigen-related MBL-2 leukaemia cell growth was greater than that of antigen unrelated P815 cells. This finding is only in part due to specific lytic activity, since untreated MLTC cells also cause growth inhibition (Figure 3, panel a), and, in part, to the higher susceptibility of MBL-2 cells to the effect of free ricin (Figure 3, panel b).

*Distribution of ricin-treated T-lymphocytes* in vivo

Numerous studies indicate that T-lymphocytes maintained in long term culture are trapped mainly in the lungs, and liver of the animals in which they are transferred (Dailey *et al.*, 1982; Carroll *et al.*, 1983). Sparshott *et al.* (1985) have shown that ricin-treated thoracic duct lymphocytes injected into normal animals behave as untreated cells as far as extravasative capacity is concerned. We deemed it necessary to determine the behaviour pattern of ricin-treated cultured T-cells following injection into animals. Therefore, $2 \times 10^6$ $^{51}$Cr-labelled MLTC cells were injected i.v. into sublethally irradiated mice, which were killed 1 or 6h later. After 1h no differences in localization between ricin-treated and untreated MLTC cells were observed (Figure 4) broadly in accord with the more extensive results of Sparshott and her colleagues. Most of the radioactivity was as expected found in the lungs, and to a lower extent in the liver. Moreover, the percentage recovery of injected radioactivity in mice receiving ricin-treated and untreated MLTC cells was similar (43% and 47%, respectively). After 6h, there was a decline in radioactivity in the lungs, accompanied by an increase in the liver; this change in the pattern of cell distribution was more pronounced when ricin-treated MLTC cells were transferred. Similar results were obtained using CTL clone cells whose recovery was evaluated 6h after injection. In this instance, the recovery was 7% in the lungs and 29% in the liver in mice injected with ricin-treated cells and 20% in the lung and 21% in the liver in mice receiving untreated cloned cells (data not shown). One group of mice was also injected with MLTC cells pre-treated with $^{125}$I-labelled ricin. In these mice after 6h a high recovery of radiolabel in lungs and liver (6% and 12%, respectively), comparable to that of mice injected with $^{51}$Cr-labelled cells, was observed. In addition, 13% of the radioactivity was detected in the gastrointestinal tract (data not shown). This is probably due to toxin freed from cells rather than cell migration because the same distribution is found when toxin alone is injected. These results also indicate that free ricin is eliminated faster than ricin interalized in the cells, since only 11% of the injected toxin was recovered after 6h in mice receiving ricin alone, while 48% of the radioactivity was recovered at this time interval in mice receiving labelled cells.

*Antitumour activity of ricin-treated T-lymphocyte transfer*

We previously observed that the transfer of virus-specific T-lymphocytes i.v. into syngeneic immunodepressed mice can prevent the development of the autochthonous tumours which otherwise develop following simultaneous injection of M-MSV at a distant site (Collavo *et al.*, 1980). This tumour model was chosen to study the effect of adoptive transfer of ricin-treated virus-specific T-lymphocytes, in view of its high sensitivity to CTL activity and because tumour size is readily evaluated.

Since several studies have demonstrated the antitumour properties of ricin (Lin *et al.*, 1970; Fodstad *et al.*, 1976, 1977; Fodstad & Pihl, 1978), preliminary experiments were carried out to evaluate its effect on M-MSV-induced tumour growth. Tumour-bearing irradiated mice, receiving M-MSV in the rear footpad 5 to 6 days previously, were injected i.v. with various ricin doses; dose-response curves of tumour growth in control and in ricin-treated mice show that tumour growth slows down only when the near lethal dose of 20ng of ricin was injected (Figure 5); 20% of mice died in 6 to 7 days.

To evaluate the therapeutic effect of ricin-treated virus-specific T-lymphocytes, MLTC cells were pre-incubated with 10$\mu$g of toxin for 1h at 37°C. After repeated washing in lactose-containing medium, the cells were further incubated at 37°C for 30min in order to eliminate ricin which is rapidly released during this period. Sublethally irradiated mice bearing M-MSV-induced tumours received an i.v. inoculum of ricin-treated MLTC cells. Mice injected with M-MSV only, or with untreated virus-specific MLTC cells, were used as controls. Following the injection of 1 or $2 \times 10^6$ ricin-treated MLTC cells the tumour stopped growing for several days, and then grew again at a slower rate (Figure 6). Transfer of $2 \times 10^6$ untreated MLTC cells had no protective effect. It should however be noted that injection of $10^7$ untreated MLTC cells can be protective. At the cell dose used, all mice survived for more than 1 month, and eventually died following tumour enlargement. When $4 \times 10^6$ treated MLTC cells were injected all mice died within 4 to 5 days due to ricin toxicity (data not shown).

In order to evaluate whether the protective effect exerted

**Figure 4** *In vivo* localization of untreated (△) or ricin treated (▲) MLTC cells in different organs of recipient mice at 1 h and 6 h after i.v. injection. Results are expressed as the percentage of injected radioactivity. Each point represents the mean (±s.d.) of 5 mice.

**Figure 5** M-MSV tumour-growth following i.v. injection of different doses of ricin into tumour bearing mice: (▲) no ricin; (●) 5 ng ricin; (○) 10 ng ricin; (□) 20 ng ricin. Broken line represents the size of uninjected footpad. Each point represents the mean (±s.d.) of 5 mice.

**Figure 6** M-MSV tumour-growth following i.v. transfer of untreated or ricin-treated cells into tumour bearing mice: (▲) no cell transfer; (○) $2 \times 10^6$ untreated virus-specific MLTC cells; (●△) $1 \times 10^6$ or $2 \times 10^6$ ricin-treated virus-specific MLTC cells; (□) $2 \times 10^6$ untreated alloreactive MLC cells; (■) $2 \times 10^6$ allo-reactive ricin-treated MLC cells. Broken line represents the size of uninjected footpad. Each point represents the mean (±s.d.) of 5 mice.

by the transferred cells was specific for M-MSV antigens, $2 \times 10^6$ lymphocytes sensitized in mixed lymphocyte culture against H-$2^d$ alloantigens were pretreated with $10\,\mu g$ of ricin and injected i.v. into tumour bearing irradiated mice. We observed that ricin-treated alloreactive cells did not inhibit M-MSV tumour growth, while virus-specific MLTC cells were efficient (Figure 6).

It was then considered that tumour growth inhibition might result from a combined effect of CTL lytic activity and ricin anti-tumour activity. Therefore, virus-specific lymphocytes, obtained in MLTC, were pretreated with anti Lyt 2 monoclonal antibodies and C to eliminate Lyt $2^+$ cytotoxic cells; the remaining Lyt $1^+2^-$ enriched cell population, which lacked lytic activity against MBL-2 lymphoma cells, was pretreated with ricin and injected i.v. into tumour bearing recipient mice. As shown in Figure 7, the transfer of the non-cytolytic T-cell fraction conferred a temporary protection provided that cells were pretreated with ricin; untreated cells were again inefficient.

Recent results indicate that it is possible to cure mice of M-MSV-induced tumours by transferring M-MSV-specific CTL clones (Engers *et al.*, 1984; Cerundolo *et al.*, 1984);

**Figure 7** M-MSV tumour-growth following i.v. transfer of untreated or ricin-treated virus-specific Lyt-$2^-$ MLTC cell fraction into tumour bearing mice: (▲) no cell transfer; (○) $2 \times 10^6$ untreated Lyt-$2^-$ MLTC cells; (●) $2 \times 10^6$ Lyt-$2^-$ ricin-treated MLTC cells. Broken line represents to the size of uninjected footpad. Each point represents the mean (±s.d.) of 5 mice.

**Figure 8** M-MSV tumour-growth following i.v. transfer of untreated or ricin-treated virus-specific CTL clone cells into tumour bearing mice: (▲) no cell transfer; (○) $0.5 \times 10^6$ untreated CTL clone; (●) $0.5 \times 10^6$ ricin-treated CTL clone. Broken line represents to the size of uninjected footpad. Each point represents the mean (±s.d.) of 5 mice.

however, a protective effect is achieved only by using large ($2 \times 10^7$) cell doses. It was thus considered interesting to study whether low numbers of ricin-treated cloned cells might be effective. We observed that the injection of $0.5 \times 10^6$ cells from a M-MSV-specific CTL clone pretreated with ricin was sufficient to inhibit tumour growth for six days (Figure 8); transfer of higher cell doses ($2 \times 10^6$) caused rapid death of the recipient mice presumably due to ricin toxicity (data not shown).

### Discussion

The initial purpose of this work was to ascertain whether CTL that had been exposed to ricin were still capable of exercising their lytic activity. The answer is yes but not for long.

Ricin is an inhibitor of ribosomal protein synthesis via the agency of its A-chain. It is believed that the B-chain of the toxin binds the holotoxin molecule to the surface of target cells prior to its internalisation partly if not entirely in coated-pit vesicles (Olsnes & Pihl, 1982). Subsequently at least one A-chain is required to enter the cytoplasm if cell death is to occur. It is known that the process of internalisation of holotoxin can take place in minutes and also that when the cells concerned have been exposed to high concentrations of toxin, inhibition of protein synthesis can be complete in two hours (Barbieri & Stirpe, 1982). It is about 48 h after this that the plasma membrane of the poisoned cell disintegrates, but there is of course loss of a number of cell functions prior to this final stage. The relatively rapid drop off in the capacity of lyse target cells, seen in the present study, is not surprising since CTL activity is impaired by numerous protein synthesis inhibitors (Henney, 1973). The small difference observed between the cloned cytotoxic cells and the MLTC cells as far as the rate of decline of cytotoxic potential after contact with ricin would require confirmation before its further discussion were worthwhile.

*In vitro* the difference between the killing effect of ricin carrying cells and those cytotoxic in their own right was evident but not large, nor perhaps could it expected to be. The conditions in which cytotoxic assays are enacted at least in the present study mean that the target cells are the only ones of which the growth inhibition can be recorded. Any ricin released from the effector cells will also have toxicity for all cells alike in the culture. Seen in this light, any selectivity of binding possessed by the effector cells was, in the circumstances of the present experiments, not likely to result in any particularly non-random distribution of the carried toxin. In this regard, ricin released by MLTC cells was able to inhibit proliferation of antigen-related MBL-2 and unrelated P815 leukaemic cells.

The results *in vivo* merit more detailed consideration. There the tumour target cells represent a relatively small part of the host animal into which the cytotoxic cells, ricin-carrying or not, were injected. The specific cytotoxicity of toxin is only that imparted to it by the effector cells. The preliminary results in the present study show, as did the more thorough studies of Sparshott and her colleagues (1985), using normal lymphocytes, that ricin carrying cells can localise in a similar manner to cells not carrying ricin at least for a short time. It should be noted that the sites of principal localisation in the present studies were not the sites of tumour growth although it must be remarked that it would be appropriate to repeat the localisation studies in tumour-bearing animals. In parenthesis it should be noted that attempts to demonstrate tumour localisation by injected cytotoxic cells (e.g. Carroll *et al.*, 1983; De Jong *et al.*, 1985) have only ever shown 2–4% of the cells injected at the site of the tumour even in circumstances in which a therapeutic effect has been observed.

In the present study it was shown that ricin is lost from the effector cells *in vitro* at a steady rate and that there is a steady decline in the capability specifically to kill. It is reasonable to assume that similar timing pertains *in vivo*. In such circumstances the demonstration of a degree of tumour inhibition without death of the host animal is encouraging.

Clearly the experiments could further be refined to optimise the effects seen. The strategy to be adopted could depend on the use of larger numbers of cells carrying smaller burdens of ricin. Such studies will be undertaken but it is apparent from the present experiments that the margin for manoeuvre between enhancement of specific cytotoxicity by ricin carrying and killing the animals due to release of toxin in inappropriate places is small.

We wish to thank Dr F. Stirpe for providing purified ricin and for stimulating discussion throughout the course of this work. We also thank Drs M. Colombatti and A. Colombatti for preparation of iodinated ricin. Mr S. Mezzalira and Mrs G. Miazzo for the competent technical assistance, Mrs P. Segato for expert help in the manuscript preparation and Mrs W. Tognon for secretarial assistance are gratefully acknowledged.

This work was supported by grants from Consiglio Nazionale delle Ricerche: Progetto Finalizzato Oncologia no.84.00523.44, Associazione Italiana per la Ricerca sul Cancro and M.P.I. 40% Programma Immunologia di Base. AJSD and DMcI were supported by grants to the Institute of Cancer Research from the Medical Research Council and the Cancer Research Campaign.

V.C. is recipient of a fellowship of Associazione Italiana per la Ricerca sul Cancro (AIRC).

### References

BARBIERI, L. & STIRPE, F. (1982). Ribosome-inactivating proteins from plants: Properties and possible uses. *Cancer Surveys*, **1**, 489.

CARROLL, A.M., PALLADINO, M.A., OETTGEN, H. & DE SOUSA, M. (1983). *In vivo* localization of cloned IL-2 dependent T-cells. *Cell. Immunol.*, **76**, 69.

CEROTTINI, J.-C. & BRUNNER, K.T. (1974). Cell mediated cytotoxicity, allograft rejection and tumor immunity. *Adv. Immunol.*, **18**, 67.

CERUNDOLO, V., ENGERS, H.D., ZANOVELLO, P., RONCHESE F. & COLLAVO, D. (1984). In *New trends in experimental hematology*. Protection from virus induced tumors by the injection of virus-specific cytotoxic T-lymphocyte clones. Peschle, C. & Rizzoli, C. (eds), p. 271. Serono: Roma.

COLLAVO, D., PARENTI, A., BIASI, G., CHIECO-BIANCHI, L. & COLOMBATTI, A. (1978). Secondary *in vitro* generation of cytolytic T-lymphocytes (CTLs) in the murine sarcoma virus system. Virus-specific CTL induction across the H-2 barrier. *J. Natl. Cancer Inst.*, **61**, 885.

COLLAVO, D., ZAÑOVELLO, P., LEUCHARS, E., DAVIES, A.J.S., CHIECO-BIANCHI, L. & BIASI, G. (1980). Moloney murine sarcoma virus oncogenesis in T-lymphocyte-deprived mice. Biological and Immunologic studies. *J. Natl. Cancer Inst.*, **64**, 97.

DAILEY, M.O., FATHMAN, G.C., BUTCHER, E.C., PILLEMER, E. & WEISSMAN, I. (1982). Abnormal migration of T-lymphocyte clones. *J. Immunol.*, **128**, 2134.

EIKLID, K., OLSNES, S. & PIHL, A. (1980). Entry of lethal doses of abrin, ricin and modeccin into the cytosol of HeLa cells. *Expl. Cell Res.*, **126**, 321.

ENGERS, H.D., LAHAYE, T., SORENSON, G.D., GLASEBROOK, A.L., HORVATH, C. & BRUNNER, K.T. (1984). Functional activity *in vivo* of effector T-cell populations. II. Antitumor activity exhibited by syngeneic anti-MoMuLV-specific cytolytic T-cell clones. *J. Immunol.*, **133**, 1664.

FODSTAD, O., OLSNES, S. & PIHL, A. (1976). Toxicity distribution and elimination of the cancerostatic lectins abrin and ricin after parenteral injection into mice. *Br. J. Cancer*, **34**, 418.

FODSTAD, O., OLSNES, S. & PIHL, A. (1977). Inhibitory effect of abrin on the growth of transplantable murine tumors and of abrin on human cancer in nude mice. *Cancer Res.*, **37**, 4559.

FODSTAD, O. & PIHL, A. (1978). Effect of ricin and abrin on survival of L1210 leukemic mice and on leukemic and normal bone-marrow cells. *Int. J. Cancer*, **22**, 558.

HENNEY, C.S. (1973). On the mechanism of T-cell mediated cytolysis. *Transplant. Rev.*, **17**, 37.

DE JONG, W.H., VAN DE PLAS, M.M.T., STEERENBERG, P.A., KRUIZINGA, W. & RUITENBERG, E.J. (1985). Selective localization of tumor-immune spleen cells at the tumor challenge site after adoptive transfer of line 10 tumor immunity in strain 2 guinea pigs. *J. Immunol.*, **134**, 2032.

LIN, J.Y., TSERNG, K.Y., CHEN, C.C., LIN, L.T. & TUNG, T.C. (1970). Abrin and ricin: new anti-tumour substances. *Nature*, **227**, 292.

MARTZ, E. (1975). Early steps in specific tumor cell lysis by sensitized mouse T-lymphocytes. Resolution and characterization. *J. Immunol.*, **115**, 261.

McINTOSH, D.P., EDWARDS, D.C. & DAVIES, A.J.S. (1984). Transfer of ricin toxicity by spleen cells. *Toxicon*, **22**, 293.

OLSNES, S. & PIHL, A. (1982). Toxic lectins and related proteins. In *The molecular actions of toxins and viruses*, Van Heyningen, S. & Cohen, P. (eds), p. 51. Elsevier: North Holland, Amsterdam.

SANDVIG, K., OLSNES, S. & PIHL, A. (1978). Binding, uptake and degradation of the toxic proteins abrin and ricin by toxin-resistant cell variants. *Eur. J. Biochem.*, **82**, 13.

SANDVIG, K. & OLSNES, S. (1979). Effect of temperature on the uptake, excretion, and degradation of abrin and ricin by HeLa cells. *Expl. Cell Res.*, **121**, 15.

SPARSHOTT, S.M., FORRESTER, J.A., McINTOSH, D.P., WOOD, C., DAVIES, A.J.S. & FORD, W.L. (1985). The carriage and delivery of substances to lymphatic tissues by recirculating lymphocytes. I. The concentration of ricin in lymphocyte traffic areas. *Immunology*, **54**, 731.

ZAGURY, D., BERNARD, J., THIERNESSE, N., FELDMAN, M. & BERKE, G. (1975). Isolation and characterization of individual functional reactive cytotoxic T-lymphocyte conjugation, killing and recycling at the single cell level. *Eur. J. Immunol.*, **5**, 818.